# Estimation of inbreeding using pedigree, 50k SNP chip genotypes and full sequence data in three cattle breeds

**DOI:** 10.1186/s12863-015-0227-7

**Published:** 2015-07-22

**Authors:** Qianqian Zhang, Mario PL Calus, Bernt Guldbrandtsen, Mogens S Lund, Goutam Sahana

**Affiliations:** Department of Molecular Biology and Genetics, Center for Quantitative Genetics and Genomics, Aarhus University, Tjele, DK-8830 Denmark; Animal Breeding and Genomics Centre, Wageningen UR Livestock Research, Wageningen, 6700 AH The Netherlands

**Keywords:** Inbreeding, Cattle, Whole-genome sequence, Runs of homozygosity

## Abstract

**Background:**

Levels of inbreeding in cattle populations have increased in the past due to the use of a limited number of bulls for artificial insemination. High levels of inbreeding lead to reduced genetic diversity and inbreeding depression. Various estimators based on different sources, e.g., pedigree or genomic data, have been used to estimate inbreeding coefficients in cattle populations. However, the comparative advantage of using full sequence data to assess inbreeding is unknown. We used pedigree and genomic data at different densities from 50k to full sequence variants to compare how different methods performed for the estimation of inbreeding levels in three different cattle breeds.

**Results:**

Five different estimates for inbreeding were calculated and compared in this study: pedigree based inbreeding coefficient (F_PED_); run of homozygosity (ROH)-based inbreeding coefficients (F_ROH_); genomic relationship matrix (GRM)-based inbreeding coefficients (F_GRM_); inbreeding coefficients based on excess of homozygosity (F_HOM_) and correlation of uniting gametes (F_UNI_). Estimates using ROH provided the direct estimated levels of autozygosity in the current populations and are free effects of allele frequencies and incomplete pedigrees which may increase in inaccuracy in estimation of inbreeding. The highest correlations were observed between F_ROH_ estimated from the full sequence variants and the F_ROH_ estimated from 50k SNP (single nucleotide polymorphism) genotypes. The estimator based on the correlation between uniting gametes (F_UNI_) using full genome sequences was also strongly correlated with F_ROH_ detected from sequence data.

**Conclusions:**

Estimates based on ROH directly reflected levels of homozygosity and were not influenced by allele frequencies, unlike the three other estimates evaluated (F_GRM_, F_HOM_ and F_UNI_), which depended on estimated allele frequencies. F_PED_ suffered from limited pedigree depth. Marker density affects ROH estimation. Detecting ROH based on 50k chip data was observed to give estimates similar to ROH from sequence data. In the absence of full sequence data ROH based on 50k can be used to access homozygosity levels in individuals. However, genotypes denser than 50k are required to accurately detect short ROH that are most likely identical by descent (IBD).

## Background

The definition of inbreeding coefficient (F) is the probability that two alleles in an individual are identical by descent (IBD) relative to a base population where all alleles are assumed unrelated [1]. Rates of inbreeding have increased as intensive selection was applied to the populations [2–7]. Increased levels of inbreeding result in increased probability that animals are homozygous for deleterious alleles [2, 8, 9]. Thus, inbred animals suffer from inbreeding depression with reduced fitness, and highly inbred animals may have considerably reduced lifespans [2, 6, 10–13]. Information on inbreeding is critical in the design of breeding program to control the increase in inbreeding levels and thereby controlling inbreeding depression in the progeny. Pedigree information has been used to calculate the estimated inbreeding coefficient as the expected probability that two alleles at a locus are IBD [14–16]. For example, Meuwissen and Luo proposed a method to estimate inbreeding coefficients based on pedigree data of large populations [17]. However, incomplete pedigrees result in erroneous estimates and an underestimation of levels of inbreeding [18]. VanRaden proposed a method to take into account unknown ancestors when estimating inbreeding coefficients, increasing the accuracy of inbreeding level estimates in incomplete pedigrees [19].

With the availability of Single Nucleotide Polymorphism (SNP) array genotyping technologies, long stretches of homozygous genotypes, known as runs of homozygosity (ROH) can be identified. ROH are believed to reflect an estimate of autozygosity on genomic level and generally identify genomic regions which are IBD [20, 21]. Theoretically, it is expected that ROH can be accurately estimated from the full sequence data, because these estimates do not suffer from sampling such as may be expected when subsets of loci, for instance 50k SNPs, are used [22–24]. The inbreeding coefficient can be calculated as the proportion of genome covered by ROH and has been shown to be more informative than the inbreeding coefficient estimated from pedigree data or other estimators because ROH strongly correlate with homozygous mutation load [25]. ROH have commonly been used to infer population history and to examine the effect of deleterious homozygotes caused by inbreeding in human populations [20, 26–29]. Long ROH reflect recent inbreeding, whereas short ROH reflect ancient inbreeding [26]. However, only a few studies have evaluated ROH in cattle populations. Ferenčaković et al. examined the effect of SNP density and genotyping errors when estimating autozygosity from high-throughput genomic data [24]. Estimates based on ROH also vary with different densities of genomic data. The minimum length of ROH that can be detected depends on SNP density [24, 30]. Recently, Purfield et al. detected ROH in a cattle population from SNP chip data to infer population history [31]. However, to estimate the “true” state of ROH, whole-genome sequences should be used rather than SNP chip data, but, to date, there are only few studies doing this in cattle [32]. With the advent of next-generation sequencing technology, whole-genome sequences have become available to examine the fine-scale genetic architecture of the cattle genome. It is now possible to investigate and compare how well different commonly used estimators of inbreeding level correlate with ROH estimated using next-generation sequence (NGS) data.

In recent years, widespread availability of genotype data enabled computation of inbreeding from the diagonals of genomic relationship matrices, i.e., the “GRM” method (F_GRM_), as a by-product of genomic selection. Similarly, using the genotypes, the inbreeding coefficient can be computed based on excess of homozygosity following Wright (1948) (F_HOM_) [33] and based on correlation between uniting gametes following Wright (1922) (F_UNI_) [1]. The objective of the present study was to compare different estimators for inbreeding coefficients calculated from pedigree, 50k SNP chip genotypes and full sequence data with estimates based on ROH, for three different dairy cattle breeds.

## Methods

### SNP genotyping and sequencing

A total of 89 bulls with a high genetic contribution to current Danish dairy cattle populations were selected for whole-genome resequencing. These included 32 Holstein (HOL), 27 Jersey (JER), and 30 Danish Red Cattle (RDC) bulls. RDC cattle are a composite breed with contributions from different red breeds, including Swedish Red, Finnish Ayrshire, and Brown Swiss [34]. Only bi-allelic variants SNPs with a *phred*-scaled quality score [35] higher than 100 were kept for analysis to ensure the quality of variants. Genotypes were extracted from whole-genome sequence (WGS) data using GATK [36] and a perl script. The sequence variants with read depth lower than 7 or higher than 30 were filtered out. In addition, 85 of the sequenced animals were genotyped with the Illumina 50k SNP assay (BovineSNP50 BeadChip version 1 or 2, Illumina, San Diego, CA). SNP genotyping and quality control were as described by Höglund et al. [37]. Among the whole genome sequenced animals, 4 animals were not genotyped with the 50k SNP chip. Their genotypes for the SNPs on the 50k chip were extracted from their whole-genome sequences. The quality of genotype calls from SNP chips is expected to be higher than that of whole-genome sequences; therefore, only sequence variants with a high quality score (phred score > 100) were included. The corresponding corrections for reverse strand calls in the sequence data were converted to Illumina calls by correcting locus calling from reverse strands in Illumina calls to maintain consistency of allele encoding between Illumina calls and sequence data. The concordance between the SNP chip and sequence data was ~97 %.

### Estimation of inbreeding

#### Using pedigree records (F_PED_)

Inbreeding coefficients for the 89 bulls were estimated using pedigree records (F_PED_). The average pedigree depth was ~8 generations ranging from 3 to 13. Average pedigree depth was 7, 8 and 9 for HOL, JER, and RDC, respectively. The method proposed by VanRaden [19] was used to compute inbreeding coefficients, which replaces unknown inbreeding coefficients by average inbreeding coefficients in the same generations. Inbreeding coefficients were calculated using the following formula [38]:

$$ {A}_{ii}={\displaystyle \sum_{j=1}^i}{L}_{ij}^2{D}_{jj}, $$

where ${A}_{ii}$ is the *i*^th^ diagonal element of the **A** matrix (pedigree relationship matrix), which is equal to the inbreeding coefficient of the *i*^th^ animal plus 1. L is a lower triangular matrix containing the fraction of the genes that animals derive from their ancestors, and D is a diagonal matrix containing the within family additive genetic variances of animals [17]. The computation for matrix elements *L*_*ij*_ and *D*_*jj*_ follows the rule of computation of the **A** matrix [17]. The detailed decomposition for computing ${A}_{ii}$ is explained by Meuwissen and Luo [17]. The analysis was conducted using *Relax2* software [39].

#### Using genotypes (F_ROH_, F_GRM_, F_HOM_, F_UNI_)

##### Sequence data

ROH were detected from sequence data using all bi-allelic variants according to the method of Bosse et al. [23]. This method was used to compute ROH for sequence data instead of PLINK because not all short ROH can be detected using PLINK for sequence data (the sliding window size in PLINK is fixed; therefore, ROH shorter than a certain length cannot be detected). The measure of homozygosity based on ROH (F_ROH_) from genomic data is defined as the total length of genome covered by ROH divided by the overall length of genome covered by SNPs or sequences as follows [20]:

$$ {\mathtt{F}}_{\mathtt{ROH}}=\frac{{\mathtt{L}}_{\mathtt{ROH}}}{{\mathtt{L}}_{\mathtt{AUTO}}}, $$

where L_ROH_ is the sum of ROH lengths and L_AUTO_ is the total length of autosomes covered by reads. The inbreeding coefficient was calculated by extracting ROH from sequence data. Three ROH estimates based on lengths were calculated from sequence data. The ROH was calculated separately by summing the ROH in different length classes: 1) based on all ROH; 2) ROH >1 Mbp; 3) ROH >3 Mbp.

In addition, three other estimates of inbreeding coefficients were calculated using sequence data (F_GRM_, F_HOM_, F_UNI_). The F_GRM_ estimate was calculated following VanRaden (2008) [40] based on the variance of the additive genotypes. F_GRM_ was derived from

$$ {\mathrm{F}}_{\mathrm{GRM}}=\frac{{\left[{x}_i-E\left({x}_i\right)\right]}^2}{h_i}-1=\frac{{\left({x}_i-2{\widehat{p}}_i\right)}^2}{h_i}-1, $$

where *p*_*i*_ is the observed fraction of the first allele at locus *i*, *h*_*i*_ = 2*p*_*i*_(1 − *p*_*i*_) and *x*_*i*_ is the number of copies of the reference allele (i.e., the allele whose homozygous genotype was coded as “0”) for the i^th^ SNP [41]. This was equivalent to estimating an individual’s relationship to itself (diagonal of the SNP-derived GRM). The F_HOM_ estimate was calculated based on the excess of homozygosity following Wright (1948) [33]:

$$ {\mathrm{F}}_{\mathrm{HOM}} = \left[\mathrm{O}\left(\# \hom \right)\hbox{-} \mathrm{E}\left(\# \hom \right)\right]/\left[1\hbox{-} \mathrm{E}\left(\# \hom \right)\right]=1-\frac{x_i\left(2-{x}_i\right)}{h_i}, $$

where O (# hom) and E (# hom) are the observed and expected numbers of homozygous genotypes in the sample, respectively [41]. The F_UNI_ estimate was calculated based on the correlation between uniting gametes following Wright (1922) [1]:

$$ {\mathrm{F}}_{\mathrm{UNI}}=\frac{x_i^2-\left(1+2{p}_i\right){x}_i+2{p}_i^2}{h_i}, $$

where *h*_*i*_ and *x*_*i*_ are the same as for F_GRM_ [41]. The calculations for these three estimates F_GRM_, F_HOM_ and F_UNI_ were computed using the option *–ibc* from GCTA software [41].

##### 50k SNP chip

ROH were detected from 50k SNP chip data using the software PLINK with adjusted parameters (–homozyg-density 1000, –homozyg-window-het 1, –homozyg-kb 10, –homozyg-window-snp 20) [23, 42]. These settings for PLINK to detect ROH in SNP data were chosen to make the detected ROH in SNP chip data and sequence data as similar as possible to enable comparisons of results when using different types of data. Genomic estimates of the inbreeding coefficient based on all ROH (F_ROH_) were calculated using the same formula as was used for the sequence data. The other three types of estimates (F_GRM_, F_HOM_, F_UNI_) were also calculated for genotypes extracted from 50k SNP chip data using the same methods as for sequence data.

Pearson’s correlation coefficients were calculated between estimates of inbreeding coefficients from each of pedigree records, 50k SNP genotypes, and whole-genome sequence variants. All correlations between different inbreeding coefficient estimators were tested within breed to determine whether they were significantly different from 0 using the R (http://www.r-project.org/) *cor* and *cor.test* functions.

### Impact of allele frequencies on estimators of inbreeding

As some estimators explicitly use allele frequencies to compute inbreeding coefficients, it is important to investigate how varying allele frequencies affect estimated inbreeding coefficients. Here, we investigated how the three different estimators change across the whole range of allele frequencies. For each genotype *x*_*i*_ (homozygous for the reference allele; heterozygous for the reference and non-reference allele; homozygous for the non-reference allele), the values can be written as a function of allele frequency *p*_*i*_, as shown in Table 1.

**Table 1:** Formula for calculating three estimators (F_GRM_, F_HOM_ and F_UNI_) for each genotype (homozygous for reference allele; heterozygous for reference and non-reference allele; homozygous for non-reference allele)

	F_GRM_	F_HOM_	F_UNI_
*x*_*i*_ = 0	${\mathrm{F}}_{\mathrm{GRM}}=\frac{3{p}_i-1}{1-{p}_i}$	F_HOM_ = 1	${\mathrm{F}}_{\mathrm{UNI}}=\frac{p_i}{1-{p}_i}$
*x*_*i*_ = 1	${\mathrm{F}}_{\mathrm{GRM}}=\frac{6{p_i}^2-6{p}_i+1}{2{p}_i\left(1-{p}_i\right)}$	${\mathrm{F}}_{\mathrm{HOM}}=1-\frac{1}{2{p}_i\left(1-{p}_i\right)}$	F_UNI_ = −1
*x*_*i*_ = 2	${\mathrm{F}}_{\mathrm{GRM}}=\frac{2-3{p}_i}{p_i}$	F_HOM_ = 1	${\mathrm{F}}_{\mathrm{UNI}}=\frac{1-{p}_i}{p_i}$

*x*_*i*_ is the number of reference allele

## Results

We used five different approaches (F_PED_, F_GRM_, F_HOM_, F_UNI_, F_ROH_) to estimate inbreeding coefficients using information from three different sources: pedigree, whole genome sequence and 50k SNP chip genotype data. There were total 11 estimates of inbreeding coefficients for each animal (Table 2). The average inbreeding coefficients estimated using different approaches and different data sets are presented in Table 2. The F_PED_ and F_ROH_ estimated from 50k data for HOL and JER are significantly higher than for RDC (*p* < 0.05). For inbreeding coefficients estimated from sequence data, F_ROH_, F_ROH>1Mb_, F_ROH>3Mb_, F_HOM_ and F_UNI_ differed significantly among breeds, being highest in JER and lowest in RDC. The mean F_ROH_ for 50k SNP chip data (0.066), and sequence data (0.19) are significantly higher than F_PED_ (0.016) (*p* < 0.01).

**Table 2:** Estimated mean (min-max) of pedigree-based inbreeding coefficient (F_PED_), GRM-based inbreeding coefficient (F_GRM_), inbreeding coefficients based on excess of homozygosity (F_HOM_), inbreeding coefficients based on correlation between uniting gametes (F_UNI_), ROH-based inbreeding coefficients (F_ROH_). F_ROH_ greater than 1 Mb, 3 Mb derived from sequence data were reported

			Mean			Range	
Inbreeding coefficients		HOL	JER	RDC	HOL	JER	RDC
	F_PED_	0.036^A^	0.018^B^	0.003^C^	0-0.100	0–0.060	0–0.013
50k SNP chip data	F_ROH_	0.066^A^	0.070^A^	0.038^B^	0.011–0.160	0.015–0.140	0.006–0.088
	F_GRM_	0.023^A^	−0.062^A^	0.345^B^	−0.162–0.683	−0.365–0.351	−0.055–0.653
	F_HOM_	−0.008^A^	−0.001^A^	−0.234^B^	−0.420–0.185	−0.227–0.147	−0.403–(−0.021)
	F_UNI_	0.013^A^	−0.031^B^	0.057^C^	−0.076–0.274	−0.121–0.063	−0.048–0.177
Sequence data	F_ROH_	0.187^A^	0.242^B^	0.118^C^	0.087–0.271	0.193–0.294	0.043–0.177
	F_ROH> 1Mb_	0.113^A^	0.162^B^	0.055^C^	0.060–0.205	0.104–0.225	0.009–0.110
	F_ROH> 3Mb_	0.070^A^	0.089^B^	0.027^C^	0.017–0.167	0.033–0.158	0–0.079
	F_GRM_	−0.108^A^	−0.122^A^	0.014^B^	−0.189–0.031	−0.179–(−0.031)	−0.244–0.34
	F_HOM_	0.069^A^	0.145^B^	−0.123^C^	−0.082–0.208	0.053–0.231	−0.408–0.061
	F_UNI_	0.028^A^	0.059^B^	−0.007^C^	−0.031–0.087	0.024–0.108	−0.054–0.055

*HOL* Holstein, *JER* Jersey, *RDC* Danish Red cattle. Significantly different means within each breed are indicated by a different superscript letter, *P*-values < 0.05

F_ROH_ estimated from sequence data is a direct and accurate estimate of the levels of homozygosity. It mostly reflects regions which were IBD on the genome; therefore, we limited our comparisons to comparing between F_ROH_ from sequence data with other estimates of F. High correlations were observed between F_ROH_ estimated from the 50k and sequence data with F_ROH>1Mb_ and F_ROH>3Mb_ from the sequence data for all three breeds (Tables 3, 4 and 5). The correlation between F_ROH_ estimated from 50k data and F_ROH>3Mb_ was higher than F_ROH_ estimated from 50k data and F_ROH>1Mb_ in JER and RDC (Tables 4 and 5). F_ROH_ was consistently positively correlated with F_HOM_ and F_UNI_, when both were computed from either 50k or sequence data in all three breeds (Tables 3, 4 and 5). A high correlation was found between F_ROH_ and F_UNI_, when both were computed from either 50k or sequence data in all three breeds (Tables 3, 4 and 5). However, for different breeds, F_HOM_ and F_UNI_ were correlated differently across different densities of genomic data. For HOL and RDC, the higher the density of genomic data used for F_UNI_, the higher the correlation was between F_UNI_ and F_ROH_ from sequence data (Tables 3 and 5). For HOL, the correlation between F_UNI_ and F_ROH_ from sequence data (0.95) was still higher than the correlation between F_ROH_ estimated from 50k SNP chip data and sequence data (0.87) (Table 3). In contrast to JER, F_HOM_ and F_UNI_ were most highly correlated with F_ROH_ estimated from sequence data (Table 5).

**Table 3:** Correlation coefficients between different estimates for inbreeding from different data sets for HOL

Correlation		F_PED_	50k SNP chip data				Sequence data					
			F_ROH_	F_GRM_	F_HOM_	F_UNI_	F_ROH_	F_ROH> 1Mb_	F_ROH> 3Mb_	F_GRM_	F_HOM_	F_UNI_
F_PED_		1	0.82**	−0.20	0.58**	0.20	0.73**	0.83**	0.84**	−0.26	0.78**	0.68**
50k SNP chip data	F_ROH_		1	−0.23	0.61**	0.15	0.87**	0.96**	0.96**	0.03	0.70**	0.88**
	F_GRM_			1	−0.83**	0.87**	−0.10	−0.13	−0.16	0.36*	−0.31	−0.0005
	F_HOM_				1	−0.44*	0.50**	0.58**	0.67**	−0.38*	0.66**	0.41*
	F_UNI_					1	0.27	0.29	0.27	0.21	0.11	0.35
Sequence data	F_ROH_						1	0.96**	0.91**	0.09	0.71**	0.95**
	F_ROH> 1Mb_							1	0.98**	0.01	0.77**	0.94**
	F_ROH> 3Mb_								1	−0.32	0.77**	0.90**
	F_GRM_									1	−0.61**	0.29
	F_HOM_										1	0.58**
	F_UNI_											1

*: significantly different from 0 at *p* < 0.05; **: significantly different from 0 at *p* < 0.01. F_PED_ is the inbreeding coefficient estimated from pedigree data. F_ROH_ is inbreeding coefficient estimated based on ROH for 50k data and for sequence data F_ROH> 1Mb_ and F_ROH> 3Mb_ are also reported. F_GRM_ is GRM-based inbreeding coefficient estimated from 50k and sequence data. F_HOM_ is inbreeding coefficient estimated based on excess of homozygosity for 50k and sequence data. F_UNI_ is the inbreeding coefficient estimated based on correlation of uniting gametes for 50k and sequence data

**Table 4:** Correlation coefficients between different estimates for inbreeding from different data sets for JER

Correlation		F_PED_	50k SNP chip data				Sequence data					
			F_ROH_	F_GRM_	F_HOM_	F_UNI_	F_ROH_	F_ROH> 1Mb_	F_ROH> 3Mb_	F_GRM_	F_HOM_	F_UNI_
F_PED_		1	0.47*	−0.18	0.46*	0.25	0.46*	0.52*	0.53*	−0.21	0.60**	0.43*
50k SNP chip data	F_ROH_		1	0.36	0.06	0.79**	0.92**	0.93**	0.96**	0.29	0.67**	0.96**
	F_GRM_			1	−0.89**	0.80**	0.19	0.16	0.22	0.86**	−0.34	0.44*
	F_HOM_				1	0.24	0.28	0.21	−0.76**	0.66**	−0.01	0.24
	F_UNI_					0.67**	0.67**	0.71**	0.69**	0.20	0.84**	0.67**
Sequence data	F_ROH_						1	0.99**	0.96**	0.14	0.76**	0.92**
	F_ROH> 1Mb_							1	0.97**	0.094	0.80**	0.91**
	F_ROH> 3Mb_								1	0.20	0.74**	0.95**
	F_GRM_									1	−0.48*	0.42*
	F_HOM_										1	0.60**
	F_UNI_											1

*: significantly different from 0 at *p* < 0.05; **: significantly different from 0 at *p* < 0.01. F_PED_ is the inbreeding coefficient estimated from pedigree data. F_ROH_ is inbreeding coefficient estimated based on ROH for 50k data and for sequence data F_ROH> 1Mb_ and F_ROH> 3Mb_ are also reported. F_GRM_ is GRM-based inbreeding coefficient estimated from 50k and sequence data. F_HOM_ is inbreeding coefficient estimated based on excess of homozygosity for 50k and sequence data. F_UNI_ is the inbreeding coefficient estimated based on correlation of uniting gametes for 50k and sequence data

**Table 5:** Correlation coefficients between different estimates for inbreeding from different data sets for RDC

Correlation		F_PED_	50k SNP chip data				Sequence data					
			F_ROH_	F_GRM_	F_HOM_	F_UNI_	F_ROH_	F_ROH> 1Mb_	F_ROH> 3Mb_	F_GRM_	F_HOM_	F_UNI_
F_PED_		1	0.54**	0.36*	−0.31	0.45*	0.49**	0.54**	0.51**	−0.21	0.37*	0.32
50k SNP chip data	F_ROH_		1	0.41*	0.35	0.80**	0.85**	0.96**	0.98**	0.08	0.21	0.77**
	F_GRM_			1	−0.66**	0.82**	0.22	0.34	0.38*	−0.36	0.43*	0.05
	F_HOM_				1	−0.10	0.40*	0.40*	0.38*	0.38*	−0.23	0.52
	F_UNI_					1	0.60**	0.76**	0.79**	−0.20	0.40*	0.46*
Sequence data	F_ROH_						1	0.93**	0.87**	0.003	0.31	0.81**
	F_ROH> 1Mb_							1	0.97**	0.010	0.29	0.79**
	F_ROH> 3Mb_								1	0.038	0.25	0.76**
	F_GRM_									1	−0.95**	0.54**
	F_HOM_										1	−0.24
	F_UNI_											1

*: significantly different from 0 at *p* < 0.05; **: significantly different from 0 at *p* < 0.01. F_PED_ is the inbreeding coefficient estimated from pedigree data. F_ROH_ is inbreeding coefficient estimated based on ROH for 50k data and for sequence data F_ROH> 1Mb_ and F_ROH> 3Mb_ are also reported. F_GRM_ is GRM-based inbreeding coefficient estimated from 50k and sequence data. F_HOM_ is inbreeding coefficient estimated based on excess of homozygosity for 50k and sequence data. F_UNI_ is the inbreeding coefficient estimated based on correlation of uniting gametes for 50k and sequence data

F_PED_ was mostly intermediately correlated with F_HOM_ and F_ROH_ estimated from 50k and sequence data. The highest correlation between F_PED_ and F_ROH_ estimated from 50k and sequence data was found in HOL (Table 3). The strongest correlation among estimators of F_ROH_ (F_ROH_ from 50k or sequence data or F_ROH>3Mb_ or F_ROH>1Mb_ from sequence data) and F_PED_ was observed between F_PED_ and F_ROH>3Mb_ from sequence data in HOL (Table 3). A moderate correlation was found between F_PED_ and F_ROH_ estimated from 50k and sequence data for JER and RDC (Tables 4 and 5).

The estimate F_GRM_ from both 50k and sequence data and F_PED_ had a correlation close to zero in all three breeds and the values were often negative (Tables 3, 4 and 5). At the same time, F_GRM_ estimated from 50k and sequence data generally showed a low correlation with other estimates except between two estimates F_GRM_ estimated from 50k and sequence data in HOL and JER, and between F_GRM_ and F_UNI_ estimated from 50k data (Tables 3 and 4).

## Discussion

**Fig. 1:**
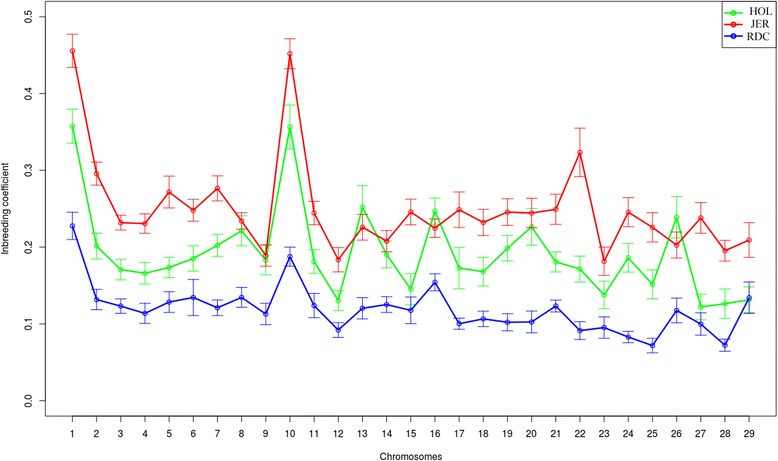
Distribution of inbreeding coefficients F_ROH_ estimated from sequence data using ROH for each chromosome in three breeds. Inbreeding coefficients F_ROH_ estimated from sequence data versus chromosomes 1–29 in HOL. JER and RDC. Standard error bars were computed among individuals within HOL, JER and RDC

Pedigree has been used to estimate inbreeding coefficients in animal breeding for over 50 years [1, 17]. Recently, researchers have utilized runs of homozygosity (ROH) estimated from medium density genotype data such as 50k SNP chip data to estimate inbreeding coefficients in livestock populations [22–24, 30]. ROH were initially used to explore regions of inbreeding in the genome and further investigate the fitness effect of these regions on different traits [2, 9, 11, 43]. Population subdivision and either inbreeding or inbreeding avoidance affects the whole genome composition, whereas selection and assortative mating will affect only those loci associated with particular phenotypes. However, we observed that inbreeding coefficient F_ROH_ estimated from sequence data were relatively higher for chromosome 1 and 10 for all four breeds (Fig. 1). This is most likely because the local recombination rate is relatively lower than average, which results in high levels of homozygosity on average [23, 44].

Our study is the first to calculate inbreeding coefficient based on ROH from full sequence data in cattle. The objective of this study was to compare estimates of inbreeding calculated from different methods and different data sources (pedigree, 50k SNP chip genotypes and full sequence data).

The pedigree-based inbreeding coefficient, F_PED_, was moderately correlated with F_HOM_ and F_ROH_ in all breeds. These moderate correlations (~0.47 to 0.56) may be partly explained by the relatively shallow depth of the pedigree records (~8–9) for these bulls. Another difference between F_ROH_ and F_PED_ is that short ROH capture ancient inbreeding while long ROH capture recent inbreeding whereas pedigree captures only relatively recent inbreeding. Pedigree accounts only for inbreeding that occurred since pedigree recording began. Therefore, after excluding ROH smaller than 1 or 3 Mbp, the correlation between F_PED_ and F_ROH_ from sequence data increased slightly for all breeds. We should also point out that a very long stretch of homozygosity using marker data might not actually be completely homozygous and therefore, higher density data was suggested to be used to detect selective sweeps through runs of homozygosity [45]. Sørensen et al. [7] has estimated inbreeding in Danish Dairy Cattle Breeds and our estimates F_PED_ are lower than theirs. This is because our sampled animals for sequencing are founder and older animals compare to the other study where they used all animals [7].

**Fig. 2:**
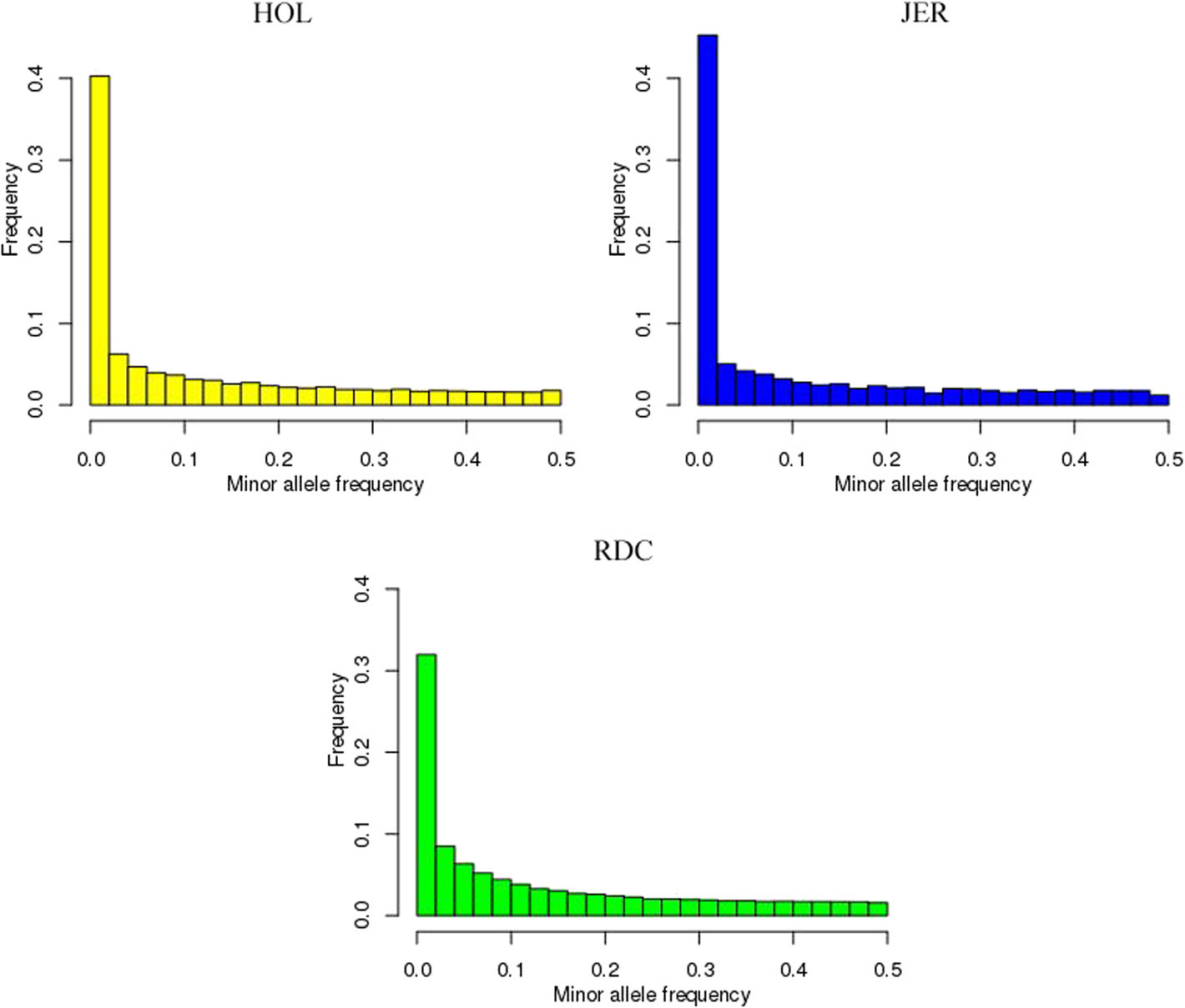
Minor allele frequency distribution for HOL, JER, and RDC bulls from sequence data. Minor allele frequency in HOL (yellow), JER (blue), and RDC (green) bulls against the minor allele frequency among all loci

Estimates of inbreeding coefficients differed with methods. Inbreeding coefficients estimates from methods using allele frequencies, i.e., F_GRM_, F_HOM_ and F_UNI_, showed considerable variation across data type and breeds. These estimators were sensitive to allele frequencies compared to ROH estimators, especially for populations with divergent allele frequencies (e.g., Fig. 2; RDC population). The estimates of genomic inbreeding coefficients are dependent on the allele frequencies in the base population [40].

**Fig. 3:**
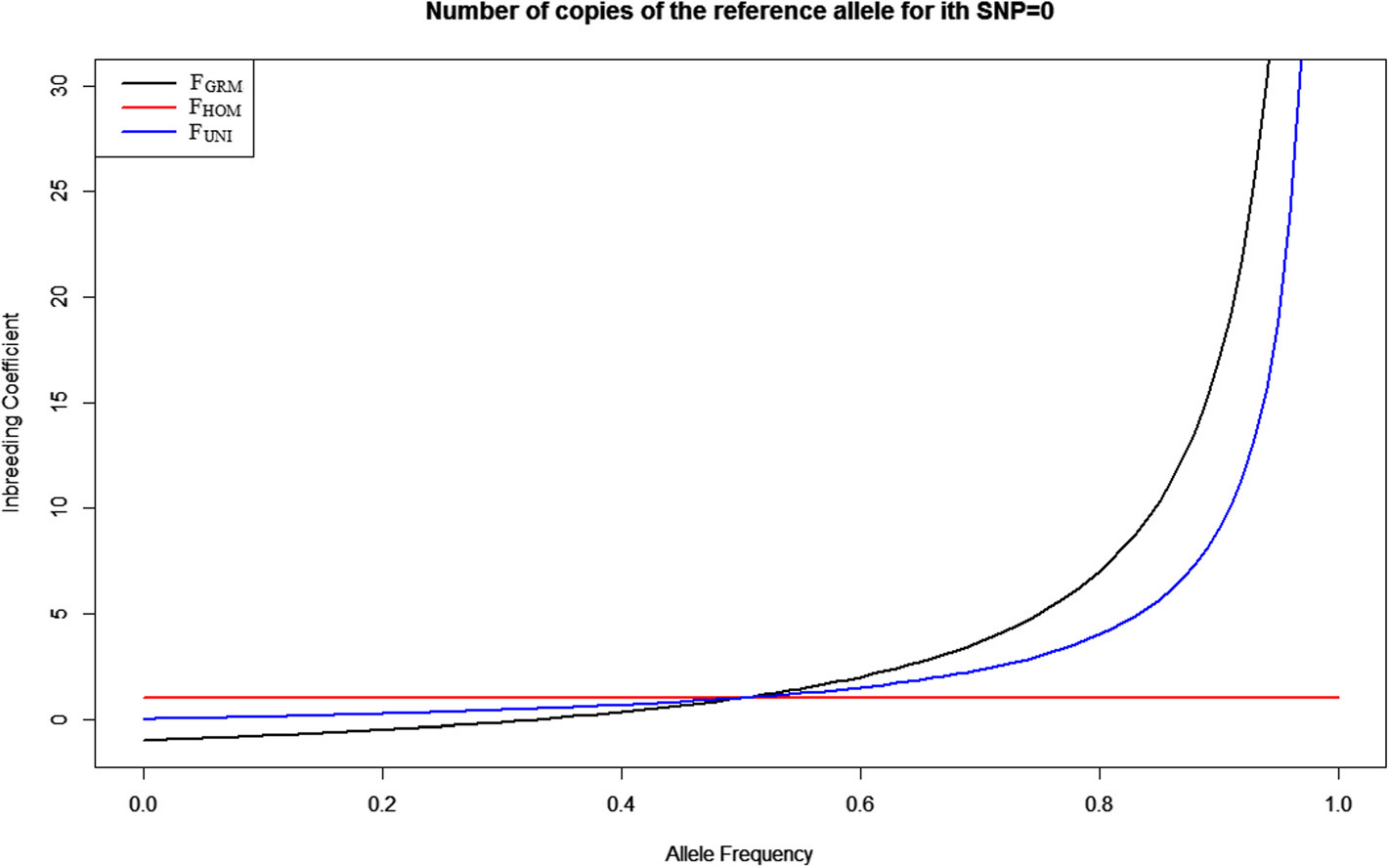
Inbreeding coefficients F_GRM_, F_HOM_ and F_UNI_ against the reference allele frequency changing from 0 to 1 when the number of copies of reference alleles for the i^th^ SNP is 0. Black line represents F_GRM_; red line represents F_HOM_ and blue line represents F_UNI_

**Fig. 4:**
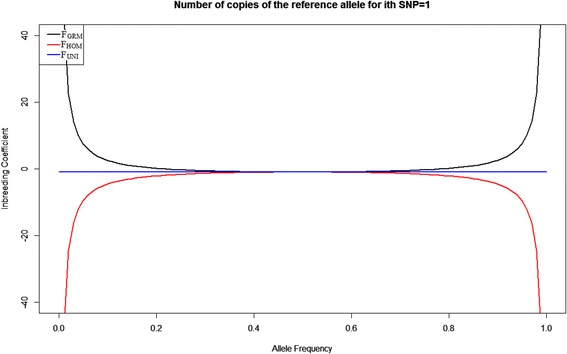
Inbreeding coefficients F_GRM_, F_HOM_ and F_UNI_ against the reference allele frequency changing from 0 to 1 when the number of copies of reference alleles for the i^th^ SNP is 1. Black line represents F_GRM_; red line represents F_HOM_ and blue line represents F_UNI_

**Fig. 5:**
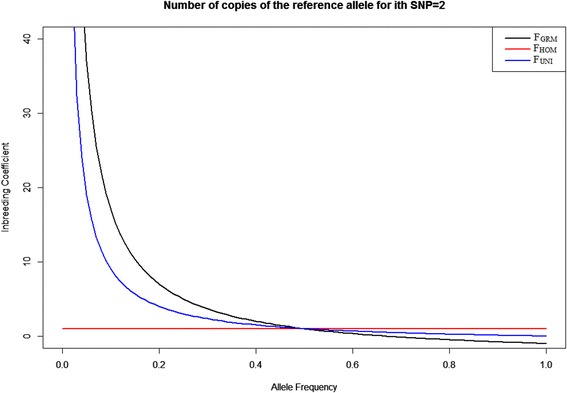
Inbreeding coefficients F_GRM_, F_HOM_ and F_UNI_ against the reference allele frequency changing from 0 to 1 when the number of copies of reference alleles for the i^th^ SNP is 2. Black line represents F_GRM_; red line represents F_HOM_ and blue line represents F_UNI_

In order to explore the reasons about the various correlations between inbreeding coefficients estimates using allele frequencies, F_GRM_, F_HOM_ and F_UNI_ were plotted against the allele frequency changing from 0 to 1 when the number of copies of reference alleles for i^th^ SNP is 0, 1 or 2 (Figs. 3, 4 and 5). When a locus is homozygous for either the reference alleles or the non-reference alleles with the allele frequency ranging from 0 to 1, F_GRM_ ranged from -1 to infinity, F_HOM_ has a constant value of 1 and F_UNI_ ranged from 0 to infinity (Figs. 3 and 5). F_HOM_ gave constant estimates for homozygous genotypes, regardless of the allele frequency (Figs. 3 and 5). When the allele frequency of the non-reference alleles is smaller than 0.2 or larger than 0.8, F_GRM_ was less than 0 (Figs. 3 and 5). When the allele frequency of the non-reference allele was between 0.2 and 0.5 or when the allele frequency of the reference allele was between 0.5 and 0.8, F_GRM_ become positive and ranges from 0 to 1 (Figs. 3 and 5).

For a heterozygous locus with an allele frequency ranging from 0 to 1, F_GRM_ and F_HOM_ ranged from minus infinity to plus infinity, and F_UNI_ has a constant value of 0 (Fig. 4). If the allele frequency was smaller than 0.2 or larger than 0.8 F_GRM_ become very large positive whereas F_HOM_ become a large negative. F_HOM_ was always negative, and F_GRM_ was always positive (Fig. 4). Thus, when a population has a high level of heterozygosity and some rare alleles with small frequency, F_GRM_ would yield large positive inbreeding coefficients, which can be misleading. This result explains why F_GRM_ was positive in the RDC breed (Table 2): this population had a higher level of heterozygosity than HOL and JER. F_UNI_ gave a stable value of 0 when the locus was heterozygous and therefore was robust to allele frequency (Fig. 4).

**Fig. 6:**
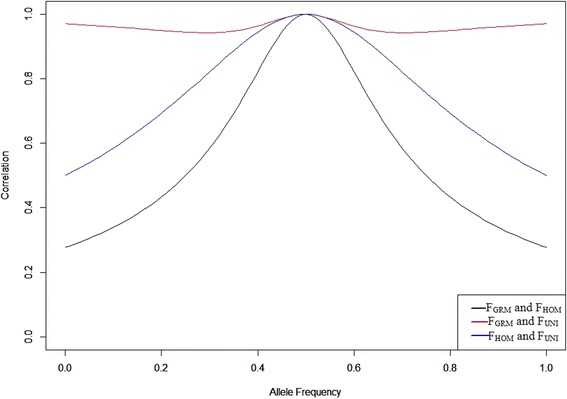
Correlations between F_GRM_ and F_HOM_, F_GRM_ and F_UNI_, and F_HOM_ and F_UNI_ when reference allele frequency changes between 0 and 1. Black line represents correlation between F_GRM_ and F_HOM_ against reference allele frequencies; red line represents correlation between F_GRM_ and F_UNI_ against reference allele frequencies and blue line represents correlation between F_HOM_ and F_UNI_ against reference allele frequencies

The correlation between the three estimators F_GRM_, F_HOM_ and F_UNI_ was computed for each of the three genotypes (i.e., homozygotes for allele 1, homozygotes for allele 2 and heterozygotes) for comparison between F_GRM_, F_HOM_ and F_UNI_ when the allele frequency was varied between 0 and 1 (Fig. 6). Correlations reached the maximal value (i.e., 1) when the allele frequencies were 0.5. When the allele frequencies were extremely high or low, correlations between estimators became low, especially the correlation between F_GRM_ and F_HOM_ (0.27). The correlation plot (Fig. 6) reflected a similar result as those in Figs. 3, 4 and 5. Therefore, when computing inbreeding coefficients using allele frequencies, populations with different allele frequencies might have very different inbreeding coefficients and the correlations between those inbreeding coefficients might be very low, with different allele frequencies.

The comparison between F_GRM_ and other estimators showed a very low correlation and F_GRM_ was mostly negatively correlated with other estimators. F_HOM_ based on excess of homozygosity was positively correlated with other estimators and was relatively highly correlated with F_ROH_ detected from 50k and sequence data. F_UNI_ based on correlations between uniting gametes estimated from 50k data generally was negatively correlated with other estimators. However, with increasing marker density, the correlation between F_UNI_ and other estimators became positive for the HOL and RDC populations. Surprisingly, when using sequence data, F_UNI_ was highly correlated with other estimators, especially F_ROH_, detected from sequence data (~0.95) for HOL. This correlation may have resulted from the nature of the estimators: F_ROH_ uses only runs of homozygosity, whereas the other estimators (to some extent) capture all of the homozygosity. This high correlation for F_UNI_ and F_ROH_ compared with low correlation between F_GRM_ and F_ROH_ might also be explained by the algorithms: F_GRM_ = (1 + F)-1 and F is the correlation between uniting gametes. This estimator has only sampling on the F-term, whereas in the F_GRM_ estimator there is also sampling variance on the “1”, which creates additional sampling variance.

It is known that RDC is an admixed breed with introgressed haplotypes from Old Danish Red, Holstein and Brown Swiss breeds. HOL and JER are relatively pure breeds and more inbred than RDC (Zhang Q, Guldbrandtsen B, Bosse M, Lund MS, Sahana G. Runs of homozygosity and distribution of functional variants in the cattle genome. BMC Genomics (in press)). Therefore, minor allele frequencies tend to be lower in HOL and JER breeds than in RDC. F_GRM_ is negatively correlated with other estimators for all three breeds. F_HOM_ becomes negative for RDC, which is likely due to the admixture present in RDC. Therefore, it appears that F_GRM_ tends to be less accurate for populations with a low minor allele frequency and that F_HOM_ tends to be less accurate for populations with a higher level of heterozygosity. This argument is supported by our results that the three inbreeding estimators F_GRM_, F_HOM_ and F_UNI_ were most closely correlated with each other when the allele frequency is approximately 0.5 (Figs. 3, 4 and 5). Therefore, the three estimators F_GRM_, F_HOM_ and F_UNI_ depend strongly on the estimation of allele frequencies in the population, unlike F_ROH_. However, here we only took one locus as an example to study the impact of allele frequencies on three estimators F_GRM_, F_HOM_ and F_UNI_.

## Conclusion

In this study, we compared different estimators of inbreeding coefficient with different types of data (pedigree, 50k SNP chip genotypes and full sequence data). Methods based on GRM, excess of homozygosity and the correlation between uniting gametes were observed to be sensitive to allele frequencies in the base population. The estimator based on pedigree data was moderately correlated with estimators based on ROH when a pedigree is relatively complete. Estimators based on ROH from SNP chip genotypes and full sequence directly reflect homozygosity on the genome, and have the advantage of not being affected by estimates of allele frequency or incompleteness of the pedigree. Inbreeding estimated from ROH was shown to be affected by the marker density used. Using sequence data, we obtained a full picture of the distribution of ROH on the genome, including short and medium length ROH that reflect ancient inbreeding regions which are possibly IBD. Detecting ROH based on high-density or 50k chip data was shown to give estimates most closely related to ROH from sequence data. However, more than 50k genotypes are required to accurately detect short ROH that are most likely identical by descent (IBD).

### Availability of supporting data

Data used in this study are from the 1000 Bull Genome Project (Daetwyler et al. 2014 Nature Genet. 46:858–865). Whole genome sequence data of individual bulls of the 1000 Bull Genomes Project are already available at NCBI using SRA no. SRP039339 (http://www.ncbi.nlm.nih.gov/bioproject/PRJNA238491).

## Abbreviations

F: Inbreeding coefficient
F_PED_: Pedigree based inbreeding coefficient
ROH: Run of homozygosity
F_ROH_: Runs of homozygosity-based inbreeding coefficients
GRM: Genomic relationship matrix
F_GRM_: Genomic relationship matrix-based inbreeding coefficients
F_HOM_: Inbreeding coefficients based on excess of homozygosity
F_UNI_: Inbreeding coefficients based on correlation of uniting gametes
IBD: Identity by descent
SNP: Single nucleotide polymorphism
NGS: Next-generation sequence
HOL: Holstein
JER: Jersey
RDC: Danish Red Cattle
WGS: Whole-genome sequence
